# Correction: Willingness and eligibility to donate blood under 12-month and 3-month deferral policies among gay, bisexual, and other men who have sex with men in Ontario, Canada

**DOI:** 10.1371/journal.pgph.0002014

**Published:** 2023-05-24

**Authors:** The PLOS Global Public Health Staff

In the author list, an author’s name was spelled incorrectly. The correct name is: JP Armstrong.

The correct citation is: Brennan DJ, Armstrong J, Kesler M, Bekele T, Lachowsky NJ, Grace D, et al. (2023) Willingness and eligibility to donate blood under 12-month and 3-month deferral policies among gay, bisexual, and other men who have sex with men in Ontario, Canada. PLOS Glob Public Health 3(1): e0001380. https://doi.org/10.1371/journal.pgph.0001380

Additionally, there are errors with this author’s contributions. JP Armstrong’s full contributions are: Conceptualization, Data curation, Formal analysis, Funding acquisition, Investigation, Methodology, Project administration, Software, Supervision, Writing–original draft, and Writing–review & editing.

The publisher apologizes for the errors.
