# Correction: Willingness and eligibility to donate blood under 12-month and 3-month deferral policies among gay, bisexual, and other men who have sex with men in Ontario, Canada

**DOI:** 10.1371/journal.pgph.0002352

**Published:** 2023-08-28

**Authors:** The PLOS Global Public Health Staff

## Notice of republication

This article was republished on August 8, 2023, to correct an author’s name. The publisher apologizes for the errors. Please download this article again to view the correct version. The originally published, uncorrected article and the republished, corrected articles are provided here for reference.

## Supporting information

S1 File Originally published, uncorrected article. (PDF)

S2 File Republished, corrected article. (PDF)
