# Supplementary material for: Willingness and eligibility to donate blood under 12-month and 3-month deferral policies among gay, bisexual, and other men who have sex with men in Ontario, Canada
Source: PLOS Glob Public Health. 2023 Jan 6;3(1):e0001380. doi: 10.1371/journal.pgph.0001380 (PMC10021805; doi:10.1371/journal.pgph.0001380)
Supplement: Attachment — Submitted filename: Brennan et al_Response to GPH Editors and Reviewers v.July28.pdf [file pgph.0001380.s001.pdf]

Response to editor and reviewer comments

PGPH-D-22-00148
Willingness and eligibility to donate blood under 12-month and 3-month deferral policies among gay, bisexual, and other men who have sex with men in Ontario, Canada.
July 28, 2022

Editor Feedback

1. You indicated that you had ethical approval for your study. In your Methods section, please ensure you have also stated whether you obtained consent from parents or guardians of the minors included in the study or whether the research ethics committee or IRB specifically waived the need for their consent.

Response: We have updated the section on study design and recruitment as a means of addressing this feedback:
"*Informed consent was obtained from all participants prior to data collection. Approval for this study was granted by the Research Ethics Boards of the University of Toronto, University of Windsor, University of Victoria, and Ryerson University. Participants who were 17 years old at the time of data collection were deemed to possess the capacity to consent to this research without approval from a parent or guardian by each Research Ethics Board.*" (lines 183-187)

2. Please amend your detailed Financial Disclosure statement. This is published with the article. It must therefore be completed in full sentences and contain the exact wording you wish to be published.
State what role the funders took in the study. If the funders had no role in your study, please state: "The funders had no role in study design, data collection and analysis, decision to publish, or preparation of the manuscript."

Response: We have amended the Financial Disclosure statement as requested:
"*This research was funded by the Canadian Blood Services' MSM Research Grant Program. The funders had no role in study design, data collection and analysis, decision to publish, or preparation of the manuscript. Additionally, David J. Brennan and Trevor A. Hart are supported by Ontario HIV Treatment Network Research Chairs in Gay and Bisexual Men's Health, JP Armstrong is supported by a Joseph-Armand Bombardier Canada Graduate Scholarship from the Government of Canada, Nathan J. Lachowsky is supported by a Scholar Award from the Michael Smith Foundation for Health Research, and Daniel Grace is supported by a Canada Research Chair in Sexual and Gender Minority Health.*"

3. Please update the Funding Information in the system to match the details with Financial Disclosure Statement like funders, grant numbers and recipients.

Response: We have included this information as requested.

4. Please provide separate figure files in .tif or .eps format.

Response: We have attached the figure files using the correct format.

5. We have amended your Competing Interest statement to comply with journal style. We kindly ask that you double check the statement and let us know if anything is incorrect.

Response: Thank you for your amendment, everything is correct.

6. In the online submission form, you indicated that your data will be submitted to a repository upon acceptance. We strongly recommend all authors deposit their data before acceptance, as the process can be lengthy and hold up publication timelines. Please note that, though access restrictions are acceptable now, your entire data will need to be made freely accessible if your manuscript is accepted for publication. This policy applies to all data except where public deposition would breach compliance with the protocol approved by your research ethics board. If you are unable to adhere to our open data policy, please kindly revise your statement to explain your reasoning and we will seek the editor's input on an exemption. Please be assured that, once you have provided your new statement, the assessment of your exemption will not hold up the peer review process.

Response: We initially believed it would have been possible to upload our data to a repository. However, the University of Toronto's Human Research Ethics Board determined that this is not possible and is willing to provide a letter stating so if necessary. Specifically, it was determined that making data publicly available would be inconsistent with our commitment to upholding privacy (as outlined in the consent form). We have updated the Data Availability statement to read:

"The datasets generated and/or analysed during the current study are not publicly available due to privacy or ethical restrictions. Please contact the corresponding author for further information."

<u>Reviewer 1 Feedback</u>

This study, conducted in Ontario, Canada, assesses the general perception among 447 men who have sex with men of eligibility and willingness to donate blood under 12 and 3 month deferral policies. The authors find the interesting correlations between increased willingness to donate and decreased age, non-White ethnicity, higher education level.

1. Table 1 shows that the region of Ontario was almost statistically significant. Could the authors briefly note this correlation in the Discussion and speak to which regions are more metropolitan, more diverse, etc.?

Response: We are hesitant to do so given that region was not statistically significant, rurality was not statistically significant, and race was not statistically significant. In our opinion, trying to link the almost statistical significance of region to the latter two factors does not seem appropriate.

2. I would strongly advise using MSM instead of GBMSM, as it is not strictly gay and bisexual men only who have sex with other men, as the authors also noted in their Methods section.

Response: The use of MSM and GBMSM is a long-standing argument in the research literature. We feel strongly about using both MSM (in reference to the policy because that is how it is named by CBS) and GBMSM (in reference to our participant base

because most define themselves as gay or bisexual and not MSM, but we prefer to be inclusive). Thank you for providing us with an opportunity to explain this choice. We have updated the short title and included an explanation for our decision to employ both terms throughout the manuscript (lines 140-146).

"These donor policies defer based on sexual behaviour (opposed to sexual identity). As such, when referring to the deferral policies we employ the term MSM. But MSM is not a label participants used to describe themselves. Consequently, we use GBMSM to describe participants as a means of respecting their diverse sexual identities. Further, the term GBMSM better captures participants' attitudes about blood donation as the deferral policy is often interpreted as discriminatory because it stigmatizes GBMSM communities and identities [14]." (lines 140-146).

3. Lines 297-299: "However, regardless of deferral period length, participants interested in blood donation were significantly more likely to indicate willingness to donate than those not interested in blood donation." – this is a circular argument, I wonder if another conclusion could be made that is more impactful.

Response: Thank you for this comment. We have reviewed the text. We view this as a description of our results and not as an argument or explanation. We have clarified this by modifying the wording (lines 310-312). See the discussion section (particularly lines 409-422) for a modified discussion of the relationship between interest and willingness.

"Under both the 12-month and 3-month deferral policies participants who were interested in blood donation were significantly more likely to indicate willingness to donate than participants who were not interested in blood donation." (lines 310-312)

"Many of the participants, who did not indicate an interest in donating blood, note the policy as a deterrent – with some specifying that they see the policy as discriminatory. The continued exclusion of MSM from blood donation, even with improved testing and ongoing monitoring and without attention to individual level risk factors, has negatively impacted how GBMSM think and feel about blood donation [1, 4, 21]. The gap in willingness between interested and non-interested participants may persist because a 3-month deferral policy—or any deferral policy singling out MSM—is still be interpreted as unjust and, thus, unacceptable [2, 21]. Indeed, Armstrong et al. [21] found that some Canadian GBMSM would only consider donating under an individual risk-based assessment applied to all donors. However, the continued difference in willingness between interested and non-interested GBMSM might also reflect the salience of other barriers noted by our participants, such as the fear of needles and blood. The extant literature similarly details fear, physical reactions, such as fainting, and a lack of convenience as detractors for some potential donors [22, 23] but reasons for donating such as altruism and self-fulfillment can outweigh these common concerns [14, 3, 24]." (lines 409-422)

4. Lines 311-314 are a bit confusing and unclear what the second part means. Please split into two sentences.

Response: Thank you. We have modified lines 324-327 to enhance clarity.

"There was a significant association between variation in the number of participants eligible to donate blood and education level (p=0.027). Specifically, a higher increase in the number of participants eligible occurred among participants who had a high school or lower level of education." (lines 324-327)

5. Lines 327-329: again, this finding seems a bit obvious. Are there any other implications of this increased willingness to donate based on increased interest?

Response: We have expanded on the implications in the discussion section (particularly lines 409-422).

"Many of the participants, who did not indicate an interest in donating blood, note the policy as a deterrent – with some specifying that they see the policy as discriminatory. The continued exclusion of MSM from blood donation, even with improved testing and ongoing monitoring and without attention to individual level risk factors, has negatively impacted how GBMSM think and feel about blood donation [1, 4, 21]. The gap in willingness between interested and non-interested participants may persist because a 3-month deferral policy—or any deferral policy singling out MSM—is still be interpreted as unjust and, thus, unacceptable [2, 21]. Indeed, Armstrong et al. [21] found that some Canadian GBMSM would only consider donating under an individual risk-based assessment applied to all donors. However, the continued difference in willingness between interested and non-interested GBMSM might also reflect the salience of other barriers noted by our participants, such as the fear of needles and blood. The extant literature similarly details fear, physical reactions, such as fainting, and a lack of convenience as detractors for some potential donors [22, 23] but reasons for donating such as altruism and self-fulfillment can outweigh these common concerns [14, 3, 24]." (lines 409-422)

6. Lines 377-379 seem a bit too speculative. Do the authors have any evidence, anecdotal or otherwise, to suggest this may be the case?

Response: We have modified lines 393-395 to remove pure speculation, add an implication of this finding and suggest an area for future research.

"This finding demonstrates that, compared to other GBMSM, gay men are disproportionately impacted by the MSM deferral policy. Differences in sex behaviour between subgroups within the GBMSM umbrella require further investigation." (lines 393-395)

7. What is the significance of having "don't remember" as one of the qualifiers in Table 1? Was that added because participants stated they did not remember? If not, please omit.

Response: Yes, there was a small number of participants who indicated that they 'don't remember' whether they have ever been interested in donating blood. Our analysis compared individuals who answered 'yes' to everyone who did not indicate an interest in donating blood (no/don't remember/prefer not to answer). This has been clarified in the methods section (lines 253-254). Further, tables and figures (Table 2, Figure 1b) were

modified ('no' was changed to 'no/don't remember/prefer not to answer') to ensure consistency.

"We also compared these numbers stratified by general interest in donation (yes vs. no/don't remember/prefer not to answer)." (lines 253-254)

<u>Reviewer 2 Feedback</u>

This study reports on the willingness and eligibility of a sub-cohort of #iCruise study participants to donate blood under 12- and 3-month deferral policies at Canadian Blood Services. Data were collected from 2018-2019. The larger study focused on sexual health outreach experiences. With 447 responses, participation in this substudy was 49%.

Overall, the study is well-designed and executed and the methodology has been previously published. One could argue that both the larger study participant group and the blood donation questionnaire exhibit self-selection bias. However, blood donors are also a highly self-selected group and this study may inform on those similarities. The statistical analyses are appropriate for the study design.

Response: Thank you for the positive comments about our study.

1. Outcome responses were not unexpected and the age group differential disinterest may be related to the implementation of the lifetime ban and those maturing under that policy. Would the authors care to speculate on that association?

Response: We have modified lines 380-390 to expand on this association.

"Previously reported data suggest that, among the general population, older adults are more likely to donate than younger adults, and at a greater frequency [18]. We find that older (50+) GBMSM are significantly more likely than their counterparts to be eligible under the 3-month deferral policy (Table 3). However, despite heightened eligibility, this age group is least likely to indicate a willingness to donate under the 3-month deferral policy (Table 2). Clackett et al. [20]. found similar differences by age among GBMSM in Australia. They suggest that older GBMSM may be less willing to donate after directly experiencing heightened stigma due to the introduction of the MSM deferral policy. Blood operators and regulators should reach out to older GBMSM when considering adjustments to their policies, as a means of better understanding this lack of willingness as well as repairing relationships." (lines 380-390)

2. Also, in light of the new Canadian Blood Service policy removing the eligibility criteria specific to MSM, how do the authors propose to address this ?

Response: The discussion section (particularly lines 409-430) was modified to reflect on the potential impact an individual risk-based assessment will have on willingness and eligibility.

"Many of the participants, who did not indicate an interest in donating blood, note the policy as a deterrent – with some specifying that they see the policy as discriminatory. The continued exclusion of MSM from blood donation, even with improved testing and

ongoing monitoring and without attention to individual level risk factors, has negatively impacted how GBMSM think and feel about blood donation [1, 4, 21]. The gap in willingness between interested and non-interested participants may persist because a 3-month deferral policy—or any deferral policy singling out MSM—is still be interpreted as unjust and, thus, unacceptable [2, 21]. Indeed, Armstrong et al. [21] found that some Canadian GBMSM would only consider donating under an individual risk-based assessment applied to all donors. However, the continued difference in willingness between interested and non-interested GBMSM might also reflect the salience of other barriers noted by our participants, such as the fear of needles and blood. The extant literature similarly details fear, physical reactions, such as fainting, and a lack of convenience as detractors for some potential donors [22, 23] but reasons for donating such as altruism and self-fulfillment can outweigh these common concerns [14, 3, 24].

Although eligibility increased significantly under the 3-month deferral policy, it is still a minority of GBMSM who are eligible to donate (Figure 1a). Blanco et al. find that an individual risk-based assessment can increase the total number of donors while maintaining the safety of the blood supply [25]. Thus, Health Canada's acceptance of an individual risk-based assessment represents a step in the right direction [15, 16]. However, future research will be needed to measure the actual impact of this policy on the eligibility (as well as the willingness) of GBMSM in Canada." (lines 409-430)

3. Page 3, lines 125-128 – the reference to policy implementation will need updating.

Response: Lines 126-131 been modified to reflect Health Canada's recent (April 2022) acceptance of an individual risk-based assessment.

"Another policy change request, seeking to remove references to sex or gender of partner from the donor questionnaire as well as employ an individual risk-based assessment applied to all donors, was put forward by the blood operator Canadian Blood Services (CBS) to the regulator Health Canada and was accepted in April 2022 [15, 16]. The implementation of this policy in September 2022 will end the blanket deferral of sexually active MSM." (lines 126-131)

<u>**Author Revisions**</u>

1. We revised the Abstract to enhance readability and provide readers with background information on the development of the MSM deferral policy in Canada (lines 48-86).

2. A section in the Background section was edited to enhance readability (lines 91-94) and some pertinent information concerning the donor questionnaire was included (lines 119-122).

3. The bisexual/other category was clarified in the Methods section (lines 231-233).

4. Following the reviewer's requested revisions, we reordered and edited a couple of paragraphs in the Discussion and Conclusion section to enhance readability (lines 361-461).

5. We edited the wording of the Acknowledgements section (lines 465-466).

6. Minor changes were made throughout the manuscript to enhance readability by addressing grammatical errors and poor word choice.

7. Edits were made to the reference section as well as the in-text citation numbering to reflect added sources.
